# The false-positive radioiodine I-131 uptake in the foreign body granuloma located in gluteal adipose tissue

**DOI:** 10.2478/v10019-011-0016-5

**Published:** 2011-06-24

**Authors:** Salih Sinan Gültekin, Alper Dilli, Ata Türker Arıkök, Hasan Bostancı, Ahmet Oğuz Hasdemir

**Affiliations:** 1 Department of Nuclear Medicine, Dışkapı Yıldırım Beyazıt Training and Research Hospital, Ankara, Turkey; 2 Department of Radiology, Dışkapı Yıldırım Beyazıt Training and Research Hospital, Ankara, Turkey; 3 Department of 1. Pathology, Dışkapı Yıldırım Beyazıt Training and Research Hospital, Ankara, Turkey; 4 Department of 2. Surgery, Dışkapı Yıldırım Beyazıt Training and Research Hospital, Ankara, Turkey; 5 Department of 1. Surgery, Dışkapı Yıldırım Beyazıt Training and Research Hospital, Ankara, Turkey

**Keywords:** thyroid cancer, false positive radioiodine uptake, post-therapy I-131 whole body scan, colour Doppler ultrasonography, magnetic resonance imaging

## Abstract

**Background:**

The purpose of using a whole-body scanning after the radioactive I-131 treatment is to screen functional residual or metastatic thyroid tissues. In whole-body scanning of some patients, false positive radioiodine I-131 uptakes may be seen in physiological uptake regions or atypical localizations.

**Case report:**

A 54 year-old woman underwent total thyroidectomy for papillary thyroid carcinoma. A positive appearance seen in the upper postero-lateral part of the right gluteal region was determined by a post-therapy I-131 whole body scan. The colour Doppler ultrasonography, magnetic resonance imaging features and histopathological characteristics of the excised lesion were presented. The lesion was demonstrated to be a foreign body granuloma.

**Conclusions:**

Unexpected positive findings in the post-therapy I-131 whole body scan should be confirmed with other imaging modalities in order to avoid unnecessary treatments. In uncertain situations, the diagnosis should be established histopathologically.

## Introduction

A total or near total thyroidectomy followed by the radioactive I-131 (RAI) treatment is administered as an initial treatment modality in selective papillary thyroid carcinoma patients.^1^ After RAI treatment, screening of functional residual or metastatic thyroid tissues is performed by a whole-body scanning (WBS). False-positive RAI uptakes may be seen in physiological uptake regions or atypical localizations where the uptake is not expected normally in varying proportions.^2^,^3^ These uptakes may sometimes be confusing and other imaging modalities and histopathological examination may be necessary in order to achieve an accurate interpretation.^4^–^11^

In our report, the patient is presented with an atypical localized RAI uptake caused by the foreign body granulom in subcutaneous fat tissue. It is an interesting case, and as far as we are aware, this is the first case of this kind in the literature.

## Case report

A 54 year-old woman was admitted to the general surgery clinic with a neck mass complaint. Thyroid gland enlargement without palpable nodularity was found on the physical examination. Neck ultrasonography revealed multiple nodules in the right thyroid lobe and there was no cervical lymphadenopathy in the clinical and ultrasonographic examination. The patient was found to be “euthyroid” in terms of thyroid functions. Fine needle aspiration biopsy of the dominant nodule was reported as “suspicious”. Total thyroidectomy was performed under general anaesthesia. In the histopathological evaluation, papillary carcinoma measuring 1 cm in diameter was determined in the right thyroid lobe. Lymphatic invasion, perineural invasion and extra capsular spread were demonstrated.

**FIGURE 1:**
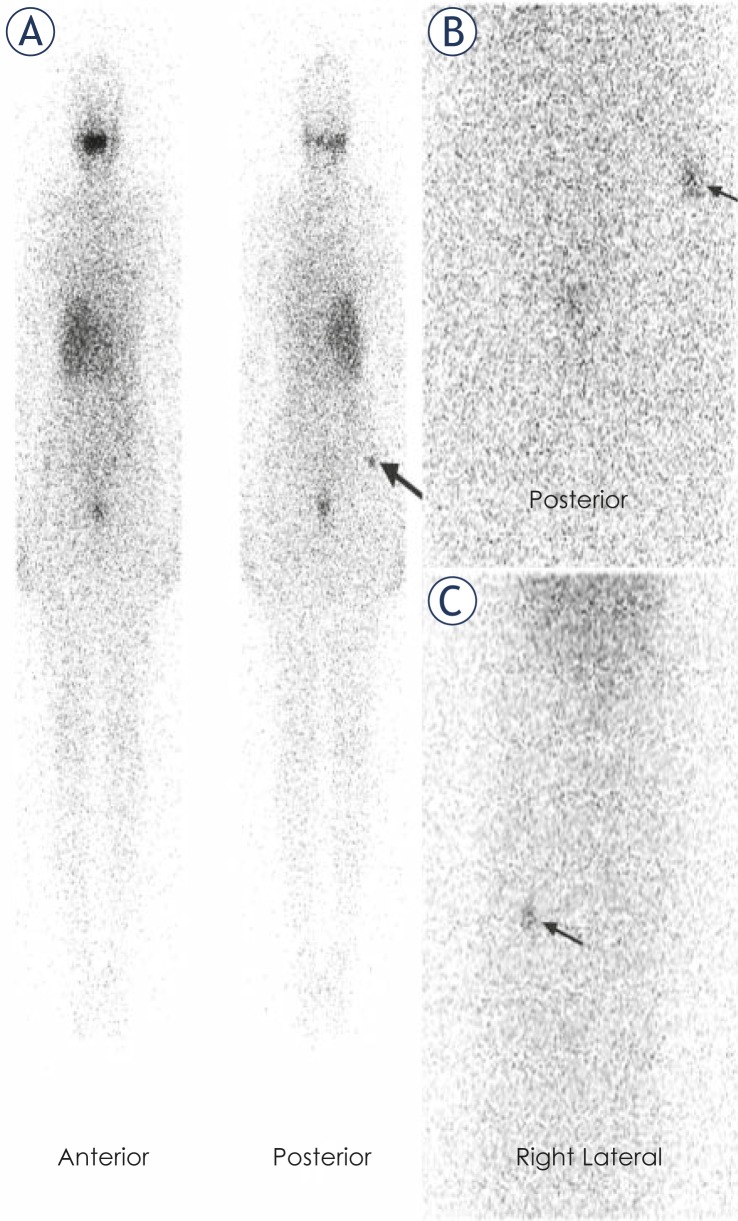
**A:** Post-therapy I-131 whole body scan performed 7 days after the administration of 5.5 GBq. Remarked focal uptake (arrow) shows upper the postero-lateral part of right gluteal region. **B** and **C:** abdominopelvic posterior and right lateral static images taken after 24 hours demonstrate stable uptake (arrows) in the same region.

**FIGURE 2:**
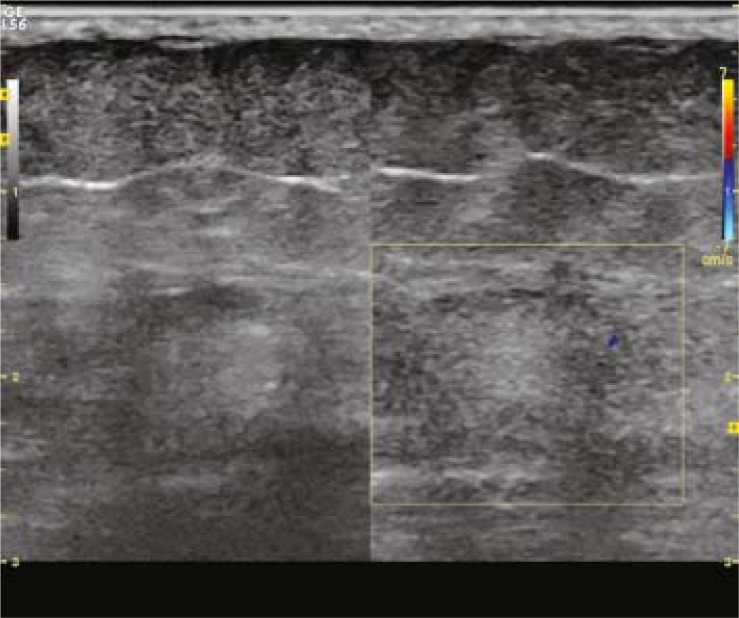
Pelvic colour Doppler ultrasound shows a lesion peripherally hypoechoic and hyperechoic in the middle with a diameter in 10 mm in the right gluteal adipose tissue. A clear blood supply example was not observed.

**FIGURE 3:**
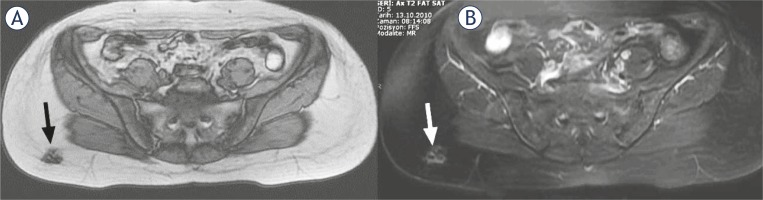
Axial magnetic resonance images. **A:** T1W image shows a hypointense lesion (black arrow) in adipose tissue in the postero-lateral part of right gluteal region. **B:** T2W fat-suppressed image shows hyperintense lesion (white arrow) with slightly irregular borders, internal structure slightly heterogeneous in the same region.

**FIGURE 4:**
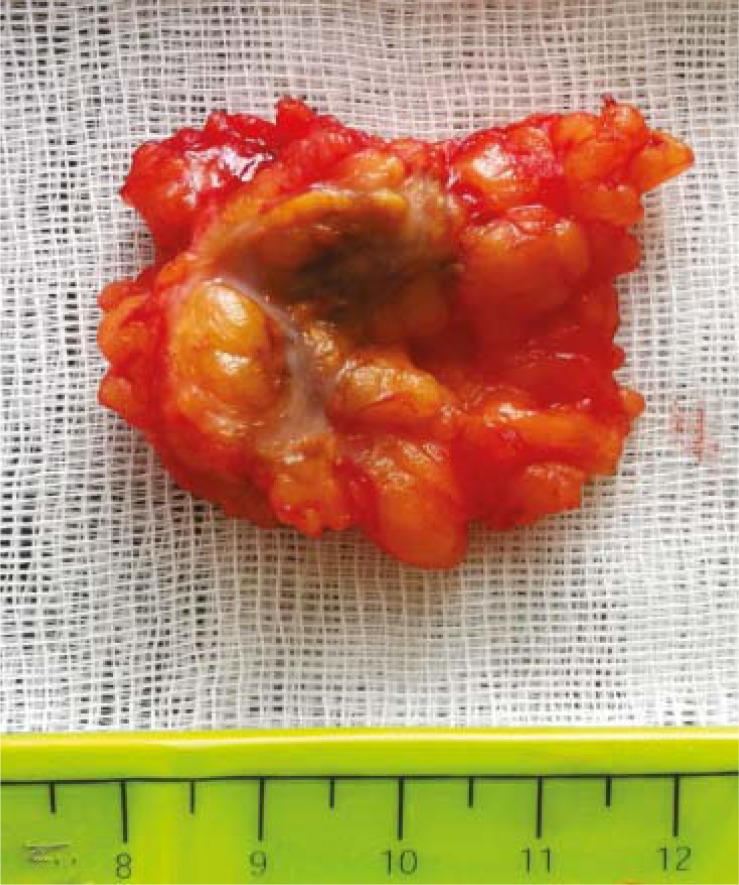
Macroscopic view of excised lesion with 3.7×2.5×2 cm dimensions. A solid region with a diameter in 1 cm in dirty cream colour separated from the other areas is shown on the cross-sectional area of the lesion.

**FIGURE 5:**
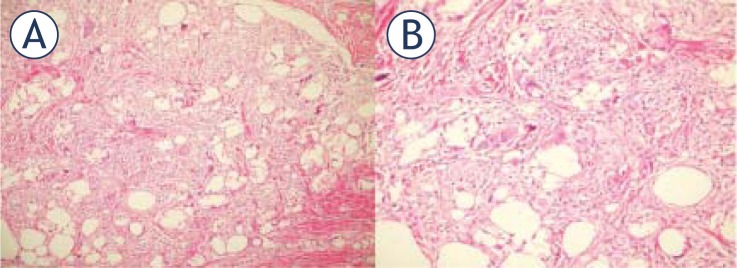
Microscopic images. **A:** Foreign body granuloma area (H&E, obj × 10). **B:** Macrophages, lymphocytes and multi-nucleated giant cells consisting of the foreign body granuloma (H&E, obj × 20).

Following total thyroidectomy the patient was not treated with thyroid hormone replacement. She was put on a low-iodine diet for four weeks. The patient was ablated with 5.5 GBq RAI when serum levels were measured as 59.5 μIU/mL for thyroid stimulating hormone, 3.69 ng/mL for thyroglobulin and 717.4 IU/ml for anti-thyroglobulin antibody. In WBS administered 7 days after ablation, the abnormal focal RAI uptake was observed in the upper postero-lateral part of the right gluteal region. The patient was advised to take a shower and wear new clothes to exclude a possible radiopharmaceutical skin contamination. On the posterior and right lateral static images received the next day, the pathological RAI uptake appeared to persist in the same region (Figure 1). In the ultrasonography, a lesion hypoechoic peripherally and hyperechoic in the middle was determined in the right gluteal adipose tissue with a diameter of 10 mm (Figure 2). The lesion did not show a clear blood supply in the color Doppler ultrasonographic examination (Figure 2). In the pelvic magnetic resonance imaging, a lesion, which was hypointense in T1-weighted images and hyperintense in T2-weighted fat-suppressed images with slightly irregular borders, was observed at the same location (Figure 3). The location of the lesion was marked with ultrasonography and was excised with safe surgical margins under local anaesthesia (Figure 4). In the histopathological examination, the lesion was found to be a foreign body granuloma (Figure 5).

## Discussion

The active transportation of iodine in follicular cells of the thyroid gland occurs *via* an “integral plasma membrane glycoprotein” called “Sodium/Iodide symporter” (NIS). NIS is known to exist and has an active role also in tissues such as salivary gland, lachrymal gland, breast tissue and gastric mucosa. Also, NIS forms the basis of cellular RAI uptake mechanism in metastatic tissues in diagnostic and therapeutic applications administered in patients with thyroid cancer.^13^ However, the role of NIS in false positive RAI uptakes is not clear.

It has been reported that false positive RAI uptakes may be seen in physiological uptake regions and atypical localizations where the uptake is not expected in general in diagnostic or post-therapy WBS. Brucker-Davis *et al.* reported false positive results in four groups as: elimination of iodine through body fluids, infection or inflammation, cyst or transudates and non-thyroid tumors.^4^ Mitchell *et al.* examined false positive RAI uptakes with similar type of tissue.^6^ Bakheet *et al.* classified false positive findings according to underlying uptake mechanisms into four groups: physiologic uptake, pathologic activity, internal retention, and external contamination by body secretions.^5^ It is predicted that leucocytes stimulate the formation of inflammatory exudates in chronic inflammatory processes or organification of iodine in leucocytes may cause the abnormal RAI accumulation.^4^,^6^ In our opinion, with the acceptance of this hypothesis more cases with any kind of chronic inflammation should be detected with WBS. This condition suggests that a different mechanism is responsible for the RAI uptake in the foreign body granuloma.

For the proper patient management, it is crucial to determine that radioactive I-131 uptakes observed outside of the neck region are real positive lesions (metastasis). In suspicious cases of false positive results, primarily, the probability of contamination or inadequate elimination of radioiodine from body fluids should be excluded.^2^–^7^ This could be excluded easily by obtaining direct late images in same or different projections (lateral, oblique) or indirect followed by applications realized for the physical decontamination or cleaning physiological uptakes. False positive uptakes that may be seen in regions where thyroid cancer metastases frequently occur (lungs, brain, skeletal system) or in other rare localizations often cause difficulties in diagnosis.^3^–^10^ In such cases, correlation with other imaging modalities and histopathological diagnosis, when possible, are necessary in order to avoid unnecessary treatments. Ultrasonography is an easy and accessible useful modality in the evaluation of soft tissue in pelvic region, but magnetic resonance modality has been found to be superior compared to other imaging modalities.^10^–^12^ The excision and the histopathological examination of lesions causing false positivity is mandatory for the definitive diagnosis.^4^,^9^

## Conclusions

This is the first report regarding the abnormal radioiodine I-131 uptake in the foreign body granuloma located in adipose tissue. Mechanism of this uptake is not clear. Further studies are recommended in order to avoid unnecessary treatments when suspicious false positive RAI uptakes exist.
